# Comparison of antimicrobial photodynamic therapy protocols as adjunct treatment in the management of initial pericoronitis: a randomized, controlled, double-blind clinical trial

**DOI:** 10.1007/s00784-026-06898-5

**Published:** 2026-05-07

**Authors:** Tânia Oppido Schalch, Ellen Sayuri Ando-Suguimoto, Bruno Souza Ferreira, Marcia Alves Pinto Mayer, Lara Jansiski Motta, Sandra Kalil Bussadori, Cinthya Cosme Gutierrez Duran, Kristianne Porta Santos Fernandes, Raquel Agnelli Mesquita-Ferrari, Christiane Pavani, Anna Carolina Ratto Tempestini Horliana

**Affiliations:** 1 Postgraduate Program in Biophotonics-Medicine, Nove de Julho University, Rua Vergueiro, 235/249 – Liberdade, São Paulo, 01504-001 Brazil; 2 Department of Microbiology, Institute of Biomedical Sciences, Cidade Universitária , University of São Paulo, CEP 05508-010 São Paulo, Brazil; 3 Postgraduate Program in Rehabilitation Sciences, Universidade Nove de Julho, Rua Vergueiro, 235/249 – Liberdade, São Paulo, 01504-001 Brazil

**Keywords:** Third molar, Pericoronitis, Antimicrobial photodynamic therapy, Laser, Randomized controlled clinical trial, Photodynamic therapy

## Abstract

**Objectives:**

The aim of this study was to compare the effectiveness of two antimicrobial photodynamic therapy (aPDT) protocols using different methylene blue formulations in the treatment of initial pericoronitis. The research question was whether the new methylene blue formulation provides superior clinical outcomes compared to the conventional formulation.

**Materials and methods:**

This randomized, controlled, double-blind clinical trial involved 34 healthy young patients with pericoronitis. The following groups were established: G1 (positive control, *n* = 17), irrigation with saline solution and aPDT with conventional methylene blue (0.005%, laser λ = 660 nm, 9 J per point, 318 J/cm²); and G2 (experimental, *n* = 17), using the same therapy but with a patented new formulation of methylene blue. Pain assessed using the Visual Analog Scale (VAS) was defined as the primary outcome. Secondary outcomes include mouth opening, edema, and quality of life (OHIP-14). Microbiological and immunological analyses were performed to complement clinical outcomes. All outcomes were assessed at baseline and on the fourth day after aPDT.

**Results:**

Both groups showed statistically significant improvement in pain (G1: *p* = 0.022; G2: *p* = 0.001) and mouth opening (G1: *p* < 0.001; G2: *p* = 0.002) after treatment. However, no statistically significant differences were observed between the groups in final pain and mouth-opening outcomes (*p* = 0.845 and *p* = 0.318, respectively).

**Conclusions:**

Within the limitations of this study, both aPDT protocols were associated with improvements in clinical outcomes. No between-group differences were observed in clinical outcomes; differences were limited to microbiological and immunological parameters, with no clinical superiority of the new formulation.

**Clinical relevance:**

Both methylene blue formulations may be used as adjunctive treatment options for the management of initial pericoronitis. However, no additional clinical benefit was observed with the new formulation, and these findings do not support a change in current clinical practice.

**Supplementary Information:**

The online version contains supplementary material available at 10.1007/s00784-026-06898-5.

## Introduction

Pericoronitis is a term used to describe inflammation of the soft tissues surrounding dental crowns and is typically observed in semi-erupted lower third molars [1].This condition constitutes the most common acute oral infection among young adults [2], with a greater frequency between the ages of 21 and 25 years [1]. Despite limited data on prevalence rates [3], studies indicate that pericoronitis is the leading reason for third molar extraction [4].

The main signs and symptoms of pericoronitis are pain, the presence of an operculum (portion of gingival tissue that partially covers the visible surface of the crown of the semi-erupted tooth), facial edema, purulent secretion, halitosis, and mucosal maceration (due to occlusal trauma from increased alveolar volume). Other commonly reported clinical manifestations include lymphadenopathy, referred pain in the ear and head, difficulty swallowing, and difficulty opening the mouth due to trismus [5].

The bacterium *Tannerella forsythia* seems to play an essential role in the development of the clinical symptoms of pericoronitis [6]. Its presence in biofilm is associated with an eight-fold increase in the risk of developing this condition compared to individuals without this bacterium in the biofilm of third molars [2]. Jakovljevic et al. (2017) [7] also report such an association, with *Tannerella forsythia* found in 40% of samples.

Despite the local and systemic harm that pericoronitis can cause, the literature on treatment is limited. Several therapies have been proposed, but there is no standardization or established gold-standard treatment [8]. This poses a challenge for the development of evidence-based therapeutic guidelines, thus complicating the clinical management of this condition.

Antimicrobial photodynamic therapy (aPDT) is a promising option that has proved to be an effective antimicrobial treatment that is easy to administer and does not induce bacterial resistance [9–11], with additional beneficial effects from both clinical and immunological standpoints. This therapy enables photobiomodulation in biological tissue [12], enhancing tissue repair and diminishing the periodontal inflammatory process. Antimicrobial PDT modulates the host’s immune-inflammatory response by interfering with inflammatory cytokine levels [12].

Previous studies [13, 14] have demonstrated the effectiveness of laser therapy in reducing postoperative pain and modulating inflammatory responses following third molar extraction. Although these findings differ from those of pericoronitis, they support the broader role of phototherapy in the management of oral inflammatory conditions.

Traditional methylene blue (MB) is widely used as a photosensitizer in dentistry and is typically formulated with water as the vehicle. Due to its planar molecular structure, MB is prone to aggregation, which can reduce light absorption and impair photodynamic efficiency [15]. Sodium dodecyl sulfate (SDS), a surfactant commonly used in oral products such as toothpastes and mouth rinses [16, 17], has been investigated as an alternative vehicle to minimize this effect. By promoting micelle formation, SDS reduces molecular aggregation, enhances the photochemical properties of MB, and may also interact with bacterial membranes, increasing permeability and facilitating antimicrobial activity [17]. Although these modifications have shown promising results, most available evidence is derived from in vitro or microbiological studies, with limited translation into clinically relevant and immunoregulatory outcomes. Therefore, well-designed randomized clinical trials are needed to evaluate the clinical applicability of SDS-based methylene blue formulations.

Using the novel MB with SDS formulation [18], reported a significant reduction in bacterial counts after five minutes of irradiation in a clinical setting. One author [16] demonstrated, in vitro, that SDS enhanced the antimicrobial efficacy of MB in aPDT by controlling molecular aggregation, highlighting the crucial role of the vehicle in photosensitizer efficiency. However, a clinical trial [19] reported no significant differences in microbial counts in periodontal pockets between aPDT using conventional methylene blue and the SDS-based formulation. These findings highlight the need for further well-designed clinical studies to assess the SDS-based methylene blue formulation.

Although modified methylene blue formulations have shown promising results, most of the available evidence comes from in vitro studies or clinical investigations. These studies are primarily focused on microbiological outcomes, without integrating clinical and immunoregulatory parameters. Furthermore, there are no randomized clinical trials specifically evaluating these formulations in pericoronitis, which limits understanding of their clinical applicability and potential impact on patient-centered outcomes.

Based on these findings, the aim of the present study was to investigate the effectiveness of aPDT in the treatment of initial pericoronitis in lower third molars. The study compared conventional methylene blue with an SDS-based formulation using microbiological, clinical, and immunoregulatory analyses. We hypothesized that the SDS-based methylene blue formulation would improve clinical, microbiological, and immunoregulatory outcomes compared to the conventional formulation in initial pericoronitis.

## Materials and methods

### Description of trial design

This is a controlled, randomized, and double-blind study. The project was approved by the Research Ethics Committee of the Universidade Nove de Julho (UNINOVE) under number 2,732,106, and it was also registered in ClinicalTrials.gov under number NCT03576105. After verbal and written explanations of the study, individuals who agreed to participate signed the Informed Consent (IC).

### Settings of data collection

The participants involved in the study were recruited from two centers: individuals who visited the Undergraduate Dental Clinic of *Universidade Nove de Julho* (UNINOVE), and those who sought screening in the Specialization Course in Oral and Maxillofacial Surgery and Traumatology (FFOUSP) for the assessment of third molars.

### Calibration

A single examiner assessed 10 individuals with pericoronitis, none of whom were part of the main study. Clinical assessment of mouth opening was performed as proposed in the present study. Intra-examiner agreement was assessed using the kappa statistic (kappa ≥ 0.87). This outcome was selected for calibration because it involves operator-dependent measurement. All clinical assessments were performed by a single examiner with more than 15 years of clinical experience. For the remaining outcomes, standardized training was conducted with the supervising researcher, who served as the reference standard, ensuring consistency in data collection.

###  Sample size calculation

**Fig. 1:**
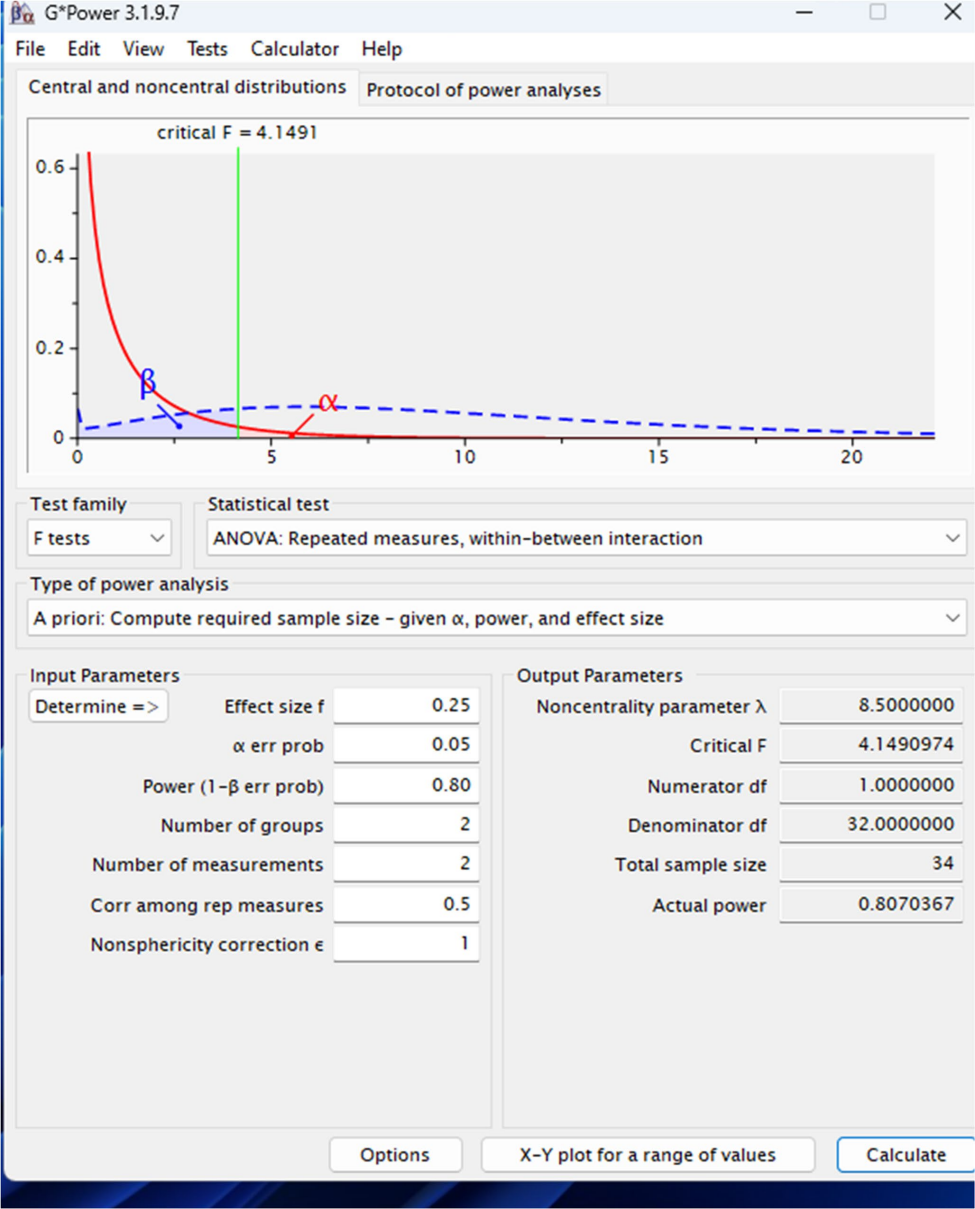
Sample size calculation

Sample size estimation was performed using G*Power version 3.1.9.7, based on a repeated-measures ANOVA model (within–between interaction), considering pain intensity (VAS) as the primary outcome. The expected difference in VAS scores was informed by Sezer et al. (2012) [20], in which the difference between the control group and the best-performing laser group at day 7 was 7.0 mm (25.0 vs. 18.0). The effect size used for sample size calculation (d = 0.4) was estimated from these data using the formula d=(M_1-M_2)/√((s_1^2 + s_2^2)/2). However, considering that both groups in the present study received active treatments, a smaller between-group difference was expected. Therefore, a more conservative moderate effect size (f = 0.25) was adopted for the sample size calculation. The calculation assumed a significance level of 5% (α = 0.05), a statistical power of 80% (1 − β = 0.80), two groups, and two repeated measurements. A correlation of 0.5 for repeated-measures data and a nonsphericity correction of 1 were adopted. Under these assumptions, the minimum required sample size was estimated at 34 participants, equally allocated between the groups (Fig. 1).

### Eligibility criteria for participants- sample description

The sample consisted of 34 healthy individuals of both sexes between 13 and 35 years of age with initial-stage pericoronitis (without fever or purulent secretion).

### Inclusion/exclusion criteria

Individuals without comorbidities between 13 and 35 years of age with an erupted or semi-erupted lower third molar with pericoronitis were included. Individuals allergic to methylene blue, those who had taken anti-inflammatory drugs or antibiotics in the previous three months, pregnant or breastfeeding women, and individuals with purulent exudate were excluded from the study.

### Randomization

An external researcher who was not involved in participant recruitment, intervention, or outcome assessment generated the random allocation sequence. The sealedenvelope.com platform was used, employing block randomization with nine blocks of four individuals. An additional six allocation slots were generated to account for potential dropouts, resulting in a total of 40 randomized allocations. Participant enrolment was conducted by the clinical research team. Allocation concealment was ensured using sequentially numbered, opaque, sealed envelopes (SNOSE), prepared by the external researcher and stored in a secure location. Each envelope was opened only at the time of treatment by a researcher not involved in outcome assessment, thereby ensuring that allocation remained concealed until the intervention was assigned.

### Blinding

Both methylene blue formulations (new formula and conventional) were prepared in advance by an external researcher. She was the only individual aware of the allocation sequence. The formulations were dispensed in identical syringes and presented identical visual characteristics (blue coloration), preventing visual identification. All clinical procedures, including irrigation, photosensitizer application, and laser irradiation parameters, were identical between groups. Consequently, neither the operator nor the participants were able to identify the allocated intervention, ensuring maintenance of the double-blind design throughout the study.

### Interventions

Thirty-four individuals were randomly assigned to two groups:

#### G1 (*n*= 17): positive control group

Subjects received irrigation with sterile saline solution followed by the application of traditional methylene blue at a concentration of 0.005%. Subsequently, photodynamic therapy was performed in a single session using a red diode laser (λ = 660 nm) operating in continuous mode, with a radiant power of 100 mW and an irradiance of 3.538 mW/cm². Irradiation was applied in contact mode at three specific sites: one vestibular, one lingual, and one in the operculum/distal sulcus region of the third molar, with an exposure time of 90 s per point (270 s total), resulting in a radiant exposure of 318 J/cm²

#### G2 (*n* = 17): experimental group

Subjects received irrigation with sterile saline solution followed by the application of a novel, patented methylene blue formulation at a concentration of 0.005% (INPI BR 1020170253902). Photodynamic therapy was then performed using the same parameters as in the control group (λ = 660 nm; continuous mode; 100 mW; 3.538 mW/cm²; 90 s per point; 318 J/cm²), applied at three sites: vestibular, lingual, and the operculum/distal sulcus region of the third molar.

### Irradiation methodology 

**Fig. 2:**
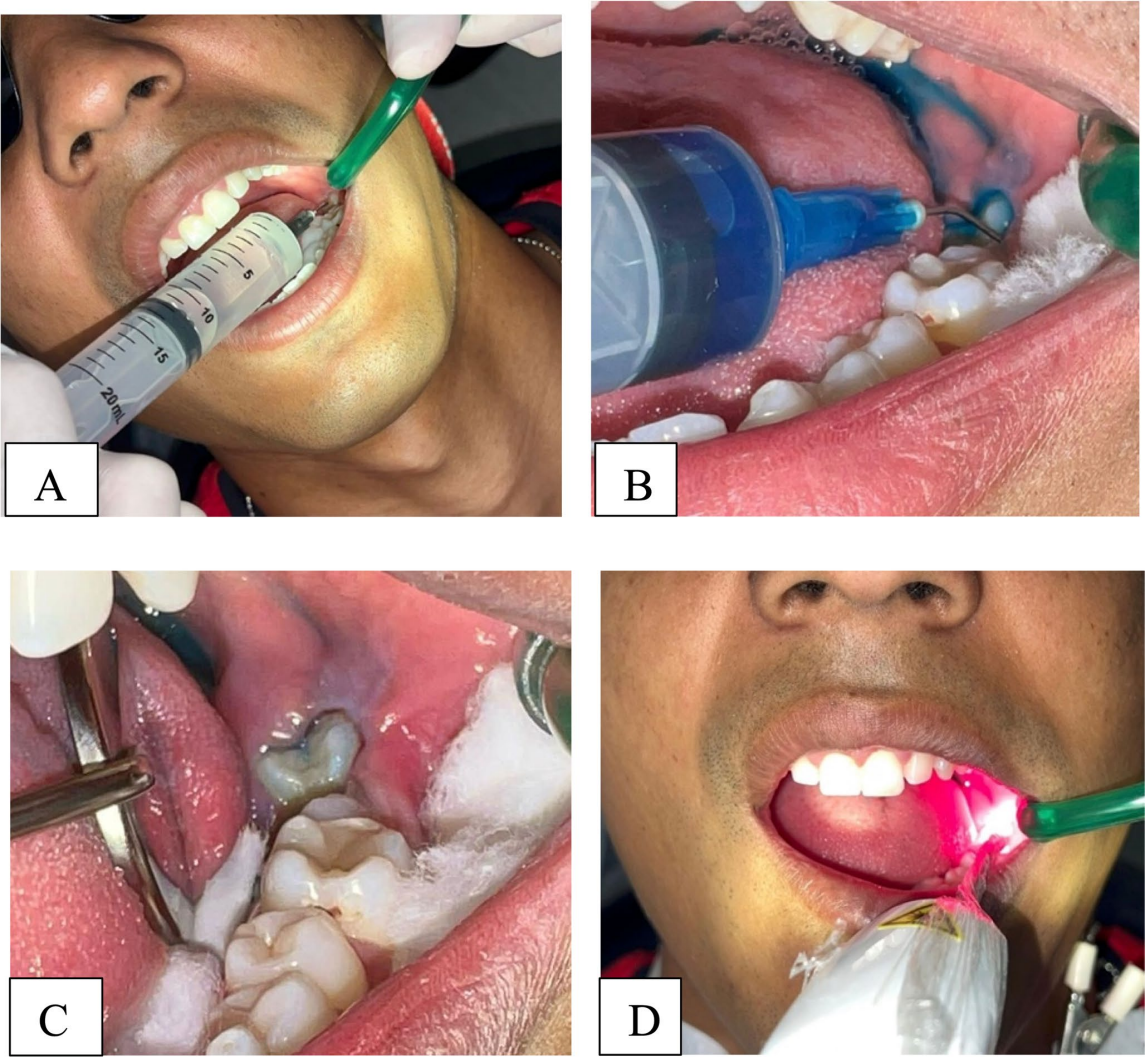
Schematic representation of the antimicrobial photodynamic therapy (aPDT) protocol sequence. Source- author’s own

 All participants (Groups 1 and 2) were irrigated with 10 mL of sterile saline solution in the operculum/gingival sulcus region of the lower third molar. Before irradiation, 0.5 mL of a 0.005% methylene blue solution was applied as a photosensitizer, consisting either of traditional methylene blue or a newly developed, patented oral-use formulation. Following a 1-minute pre-irradiation period, laser irradiation was performed perpendicular to the tissue using red light (λ = 660 nm ± 10 nm) (Therapy XT, DMC, São Carlos, Brazil; ANVISA 800308110157). The irradiation parameters, application sites, and energy delivery protocol were identical to those described for each group (Table 1). Irradiation was applied at three anatomical sites (vestibular, lingual, and operculum/distal sulcus region) (Fig. 2) [21].

**Table 1:** Parameters of antimicrobial photodynamic therapy (aPDT) used in the study

Parameter	Value
Photosensitizer	Methylene blue (0.005%)
Formulations	Conventional MB / MB + SDS
Pre-irradiation time	60 s
Wavelength	660 nm (± 10 nm)
Laser type	Red diode laser
Device	Therapy XT (DMC, São Carlos, Brazil; ANVISA 800308110157)
Power output	100 mW
Irradiance	3.538 mW/cm²
Energy per point	9 J
Irradiation time	90 s per point
Total irradiation time	270 s
Radiant exposure (fluence)	318 J/cm²
Application mode	Contact, perpendicular to tissue
Number of points	3 (vestibular, lingual, operculum/distal sulcus)
Irrigation	10 mL sterile saline
Volume of photosensitizer	0.5 mL
Number of sessions	Single session

Abbreviations: *MB* methylene blue, *SDS *sodium dodecyl sulfate, *nm *nanometers, *mW* milliwatts, *cm*², square centimeters, *J* joules, *s* seconds

### Study outcome variables - primary outcome variable: pain 

**Fig. 3:**
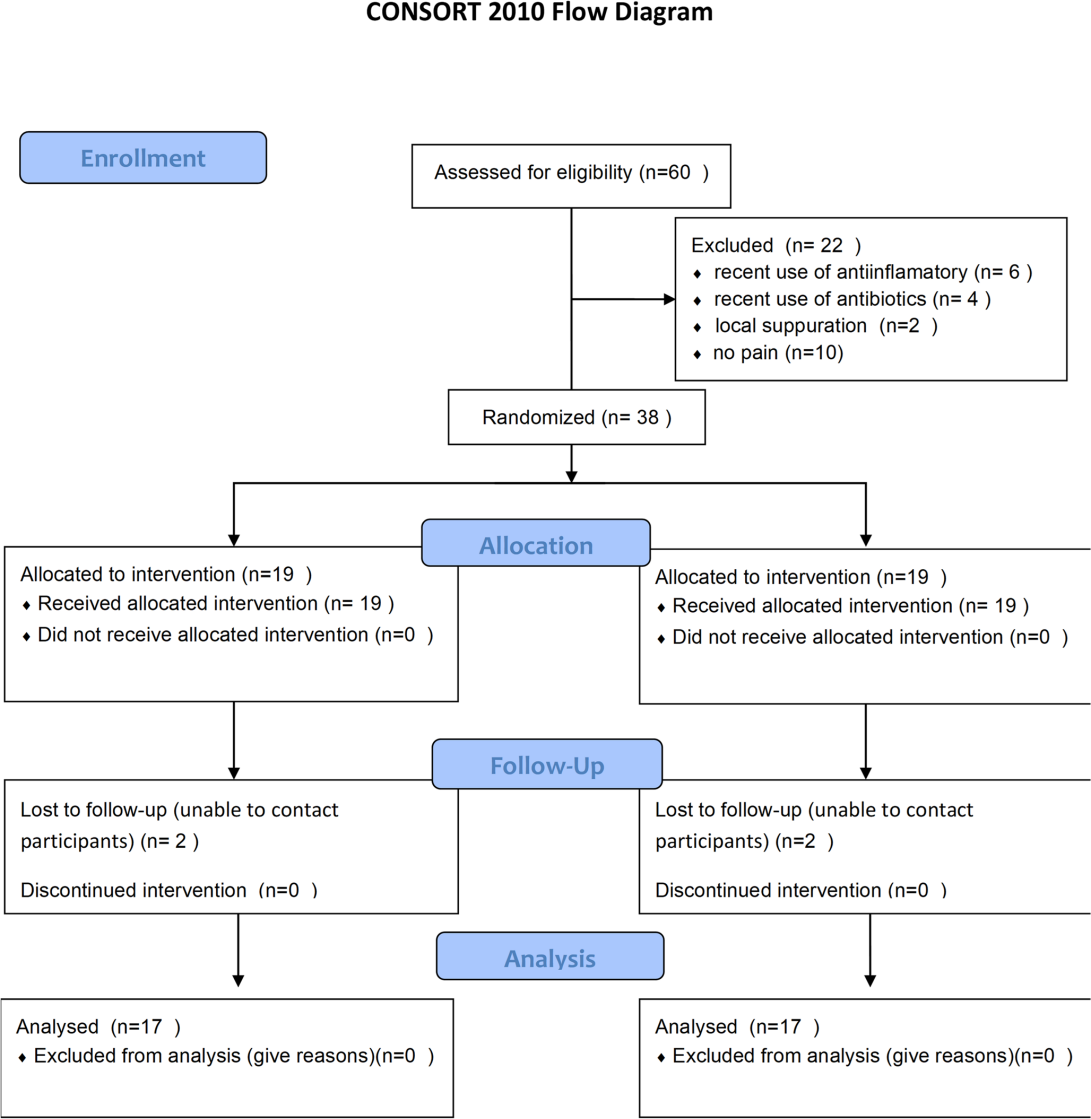
Flow chart of the study

 Pain intensity was assessed using the Visual Analog Scale (VAS), which consists of a 10-cm horizontal line marked at each centimeter and with anchored endpoints. One end was labeled 0, corresponding to “no pain,” and the other end was labeled 100, representing “worst possible pain”. The participants were instructed to indicate their pain intensity by marking a vertical line at the point on the scale that best corresponded to their perceived level of pain [22]. All the outcomes are described in a CONSORT Flowchart (Fig. 3).

### Study outcome variables - secondary outcome variables

#### Mouth opening

Measured using digital calipers for the determination of the interincisal distance between the upper and lower right central incisors [22].

#### Edema

Measured as the sum of three predetermined facial distances using a flexible ruler: (I) from the outer canthus of the eye to the angle of the mandible; (II) from the tragus to the labial commissure; and (III) from the tragus to the pogonion [23].

### Impact of oral health on well-being and quality of life

The impact of oral health on the well-being and quality of life of individuals diagnosed with pericoronitis was investigated using the Oral Health Impact Profile (OHIP-14) questionnaire. This instrument assesses seven dimensions: functional limitation, physical pain, psychological discomfort, physical disability, psychological disability, social disability, and handicap. Both the total score, calculated by summing all domains, and a partial score derived from the sum of the physical pain and physical disability domains were considered for analysis.

### Salivary cytokine and gingival crevicular fluid profiles

Saliva and gingival crevicular fluid (GCF) samples were collected prior to biofilm sampling. Unstimulated saliva samples were obtained and stored at − 80 °C until analysis.

GCF was collected from the operculum/distal sulcus region using sterile paper cones after isolation and gentle air-drying of the area, following standardized procedures adapted from Kinney et al. (2014 [24]). Samples contaminated with blood were discarded, and collection was repeated when necessary.

GCF samples were processed under standardized laboratory conditions and stored at − 80 °C until analysis. Levels of inflammatory markers (TNF-α, IL-1β, IL-6, IL-8, IL-10, IL-12, and IL-17) were quantified using a Luminex^®^ system with commercial kits (R&D Systems, MN, USA), according to the manufacturer’s instructions.

### Microbiological analysis

 Biofilm samples were collected from three sites on the mandibular third molars (disto-buccal sulcus, disto-lingual sulcus, and operculum/distal sulcus) using Mini Five™ periodontal curettes. Samples were transferred into microtubes containing Tris-EDTA buffer and stored at − 80 °C until analysis. Unstimulated saliva samples were also collected and stored under the same conditions. DNA extraction was performed using a commercial kit (Meta-G-Nome™ DNA Isolation Kit, Epicentre Technologies Corp., Chicago, IL, USA), according to the manufacturer’s instructions. DNA concentration and purity were assessed by spectrophotometry (NanoDrop ND1000, Thermo Fisher Scientific Inc.), and only samples within the acceptable range were included in the analysis. Quantitative real-time PCR (qPCR) was used to assess total bacterial load (universal 16 S rRNA primers) and the presence of *Tannerella forsythia*. Species-specific primers were used as previously described [25]. Reactions were performed using SYBR Green chemistry (Thermo Fisher Scientific, Applied Biosystems, Foster City, CA, USA) under standard conditions, and only assays with amplification efficiencies between 90% and 110% and R² values close to 1 were accepted.

As inflammatory responses are typically resolved within 2–3 days, all clinical assessments and sample collections were performed on the fourth day after aPDT to capture post-treatment outcomes (Table 2).

**Table 2:** Primer sequences used for bacterial detection by quantitative PCR

Bacterium	Primer	Sequence (5′–3′)	Reference
*Tannerella forsythia*	Forward	GGGTGAGTAACGCGTATGTAACCT	Shelburne et al., 2000
	Reverse	ACCCATCCGCAACCAATAAA	
Universal (16 S rRNA)	Forward	CCATGAAGTCGGAATCGCTAG	Shelburne et al., 2000
	Reverse	GCTTGACGGGCGGTGT	

Primer sequences used for PCR amplification of bacterial DNA. Sequences are presented in the 5′–3′ direction. Universal primers target conserved regions of the bacterial 16S rRNA gene

## Statistical methods

### Statistical analyses

Were performed with the aid of the SPSS 29 for Windows software package (SPSS, Chicago, USA). The Shapiro-Wilk test was used to assess the data’s normality. Non-parametric data (saliva and biofilm microbiological data and cytokine data) were log-transformed prior to analysis due to the inherent variability and skewed distribution of quantitative PCR data. Analyses were performed using log-transformed values to ensure appropriate distribution and variance stabilization. After logarithmic transformation, normality was reassessed using the Shapiro–Wilk test, confirming that the data met the normality assumption.

A two-way repeated measures ANOVA was performed using the General Linear Model in SPSS, with time (baseline and day 4) as a within-subject factor and treatment group as a between-subject factor, with Bonferroni post hoc adjustment.

Normally distributed data were expressed as mean ± standard deviation for the descriptive analysis. A p-value of < 0.05 was considered statistically significant. Additionally, 95% confidence intervals were calculated using SPSS, thus enabling a clearer visualization of the magnitude and direction of the effects. The analysis was conducted on a per-protocol basis, excluding participants who did not attend the follow-up assessment. No imputation of missing data was performed. The number of dropouts and reasons for loss to follow-up are reported in the study flow diagram.

## Results

Throughout the clinical collections, four participants did not return and were therefore excluded from the study. To reach the final sample size of *n* = 34, a total of 38 individuals were enrolled, following the pre-established randomization protocol. During screening, 22 patients were excluded from the study: six due to recent use of anti-inflammatory drugs, four due to recent use of antibiotics, two due to the presence of local suppuration, and 10 due to little or no pain. A CONSORT flow diagram illustrating participant recruitment, exclusion, randomization, and follow-up has been included. The study protocol was conducted from November 2020 to May 2024.

Mean age was 26.05 ± 5.02 years in the group treated with the traditional anti-inflammatory formulation and 26.52 ± 3.89 years in the group that received the novel anti-inflammatory formulation. The categorical variables “participant sex” and “treated region” are also described in Table 3.

**Table 3:** Demographic characteristics and treated region according to study groups

Variable	Traditional MB (*n* = 17)	New MB formulation (*n* = 17)
Age (years), mean ± SD	26.05 ± 5.02	26.52 ± 3.89
Sex, n (%)		
Female	12 (70.6)	11 (64.7)
Male	5 (29.4)	7 (35.3)
Treated region, n (%)		
Tooth 38	8 (47.1)	10 (58.8)
Tooth 48	9 (52.9)	7 (41.2)

Data are presented as mean ± standard deviation or number (percentage). No statistically significant differences were observed between groups (*p* > 0.05)

Regarding the primary outcome, both groups exhibited a significant reduction in pain intensity after treatment with aPDT (*p* = 0.022 in the group that received the traditional MB and *p* = 0.001 in the group that received the novel MB formulation). However, no statistically significant differences were found between groups in pain scores at baseline (*p* = 0.151) or after treatment (*p* = 0.845) (Tables 4 and 5).

**Table 4:** Comparison of clinical outcomes between groups at baseline and day 4

Outcome	Time point	Traditional MB (mean ± SD)	New MB formulation (mean ± SD)	*p*-value	95% CI
Pain (VAS)	Baseline	32.60 ± 17.98	36.80 ± 23.65	0.151	−6.81 to 30.81
	Day 4	8.27 ± 10.87	4.00 ± 6.42	0.845	−0.53 to 6.62
Mouth opening (mm)	Baseline	44.59 ± 9.46	43.26 ± 7.95	< 0.001	−3.72 to 6.02
	Day 4	50.38 ± 6.39	47.84 ± 7.25	0.318	−2.72 to 7.80
Edema (mm)	Baseline	355.41 ± 12.07	356.52 ± 16.43	0.800	−8.07 to 10.31
	Day 4	355.42 ± 11.98	356.41 ± 16.59	0.822	−8.25 to 10.25

Data are presented as mean ± standard deviation. VAS, Visual Analog Scale. Mouth opening was measured as interincisal distance (mm). Edema was assessed using standardized facial measurements. p-values refer to intergroup comparisons. CI, confidence interval

**Table 5:** Intragroup comparison of clinical outcomes between baseline and day 4

Outcome	Group	Baseline (Mean ± SD)	Day 4 (Mean ± SD)	*p*-value	95% CI
Pain (VAS)	Traditional MB	32.60 ± 17.98	8.27 ± 10.87	0.022	6.58–49.21
	New MB formulation	36.80 ± 23.65	4.00 ± 6.42	0.001	30.95–48.93
Mouth opening (mm)	Traditional MB	44.59 ± 9.46	50.38 ± 6.39	< 0.001	45.23–52.24
	New MB formulation	43.26 ± 7.95	47.84 ± 7.25	0.002	2.02–7.12
Edema (mm)	Traditional MB	355.41 ± 12.07	355.42 ± 11.98	1.000	–
	New MB formulation	356.52 ± 16.43	356.41 ± 16.59	0.735	0.607–0.842

Data are presented as mean ± standard deviation. Pain was assessed using the Visual Analog Scale (VAS). Mouth opening was measured as interincisal distance (mm). Edema was assessed using standardized facial measurements. p-values and 95% confidence intervals were calculated using two-way ANOVA with Bonferroni adjustment for multiple comparisons. Statistical significance was set at *p* < 0.05

For the secondary outcome of mouth opening, both groups also demonstrated a statistically significant improvement following the intervention (*p* < 0.001 in the traditional MB group and *p* = 0.002 in the novel formulation group). A statistically significant difference in mouth opening was observed between groups at baseline (*p* < 0.001), indicating a baseline imbalance in this variable that should be considered when interpreting the results. After treatment, no significant difference between groups was observed (*p* = 0.318) (Tables 4 and 5).

In the assessment of facial edema, no statistically significant changes were observed after aPDT in either the traditional MB group (*p* = 1.0) or the novel MB formulation group (*p* = 0.735). Moreover, no significant differences were found between the groups regarding baseline and final edema values (*p* = 0.800 and *p* = 0.822, respectively) (Tables 4 and 5).

Statistical analysis of the total OHIP score (all domains) revealed no significant differences between groups before or after treatment (*p* = 0.096) or within groups (traditional MB: *p* = 0.059; novel MB formulation: *p* = 0.600). Similarly, no significant differences were found in the partial OHIP score (only physical pain and physical disability domains) between groups (*p* = 0.691) or within groups (traditional MB: *p* = 0.329; novel MB formulation: *p* = 0.307). Tables 6 and 7 summarize the values obtained.

**Table 6:** Comparison of total and partial OHIP scores between groups at baseline and day 4

Outcome	Time point	Traditional MB (Mean ± SD)	New MB formulation (Mean ± SD)	*p*-value	95% CI
Total OHIP	Baseline	20.12 ± 8.90	23.41 ± 9.89	0.226	2.25–8.84
	Day 4	18.35 ± 8.50	22.76 ± 10.80	0.096	0.87–9.70
Partial OHIP	Baseline	9.06 ± 3.28	9.59 ± 3.65	0.612	1.63–2.69
	Day 4	8.35 ± 3.04	8.88 ± 4.35	0.691	2.24–3.29

Data are presented as mean ± standard deviation. p-values and 95% confidence intervals were calculated using two-way ANOVA with Bonferroni correction for multiple comparisons. Statistical significance was set at *p* < 0.05

**Table 7:** Intragroup comparison of total and partial OHIP scores between baseline and day 4

Outcome	Group	Baseline (Mean ± SD)	Day 4 (Mean ± SD)	*p*-value	95% CI
Total OHIP	Traditional MB	20.12 ± 8.90	18.35 ± 8.50	0.059	0.76–3.70
	New MB formulation	23.41 ± 9.89	22.76 ± 10.80	0.600	1.91–3.21
Partial OHIP	Traditional MB	9.06 ± 3.28	8.35 ± 3.04	0.329	0.77–2.19
	New MB formulation	9.59 ± 3.65	8.88 ± 4.35	0.307	0.71–2.12

Data are presented as mean ± standard deviation. p-values and 95% confidence intervals were calculated using two-way ANOVA with Bonferroni correction for multiple comparisons. Statistical significance was set at *p* < 0.05

Although the cytokines TNF-α, IL-1β, IL-6, IL-8, IL-10, and IL-17 from the gingival crevicular fluid (GCF) were submitted to analysis using the Luminex^®^ method, only IL-1β and IL-8 had detectable and relevant concentrations. Statistical analysis revealed no significant differences between the two groups at either time point for either cytokine, except for IL-1β at baseline, for which a statistically significant difference was found between groups (*p* = 0.009).

However, the intragroup analysis revealed a significant reduction in IL-1β after treatment in the group that received the novel MB formulation (*p* = 0.001), which was not observed in the traditional MB group (*p* = 0.252). No significant intragroup differences were found for IL-8 levels after aPDT. Tables 8 and 9 summarize these findings.

**Table 8:** Comparison of cytokine concentrations between groups at baseline and day 4

Cytokine	Time point	Traditional MB (pg/mL, Mean ± SD)	New MB formulation (pg/mL, Mean ± SD)		*p*-value	95% CI
IL-1β	Baseline	563.71 ± 534.29		716.25 ± 934.12	0.009	178.86–943.26
	Day 4	466.32 ± 396.52		310.79 ± 212.23	0.383	228.17–539.23
IL-8	Baseline	204.94 ± 168.67		208.18 ± 152.45	0.970	188.87–195.35
	Day 4	204.80 ± 186.71		187.85 ± 138.43	0.816	143.51–177.42

Cytokine concentrations are presented as mean ± standard deviation (pg/mL). p-values and 95% confidence intervals were calculated using two-way ANOVA with Bonferroni correction for multiple comparisons. Statistical significance was set at *p* < 0.05

**Table 9:** Intragroup comparison of cytokine concentrations between baseline and day 4

Cytokine	Group	Baseline (pg/mL, Mean ± SD)	Day 4 (pg/mL, Mean ± SD)	*p*-value	95% CI
IL-1β	Traditional MB	563.71 ± 534.29	466.32 ± 396.52	0.252	82.58–277.38
	New MB formulation	716.25 ± 934.12	310.79 ± 212.23	0.001	156.23–460.04
IL-8	Traditional MB	204.94 ± 168.67	204.80 ± 186.71	0.997	90.33–90.61
	New MB formulation	208.18 ± 152.45	187.85 ± 138.43	0.757	123.69–164.36

Cytokine concentrations are presented as mean ± standard deviation (pg/mL). p-values and 95% confidence intervals were calculated using two-way ANOVA with Bonferroni correction for multiple comparisons. Statistical significance was set at *p* < 0.05

In the analysis of *T. forsythia* in saliva, no significant differences were found between groups at baseline or four days after treatment (*p* = 0.581 and *p* = 0.123, respectively). Moreover, no significant intragroup differences were found (*p* = 0.621 and *p* = 0.881). In the biofilm samples, no significant differences were found between groups at baseline or after four days (*p* = 0.442 and *p* = 0.806, respectively) and no within-group differences were found (*p* = 0.538 and *p* = 0.518) regarding the number of *T. forsythia* 16 S rRNA gene copy pairs per µL. These data are summarized in Tables 10 and 11.

**Table 10:** Comparison of *T. forsythia 16 S rRNA* gene copy numbers between groups at baseline and day 4

Sample	Time point	Traditional MB (copies/µL, Mean ± SD)	New MB formulation (copies/µL, Mean ± SD)	*p*-value	95% CI
Saliva	Baseline	1.57 × 10³ ± 1.09 × 10³	1.31 × 10³ ± 1.26 × 10³	0.581	718.23–1224.20
	Day 4	1.88 × 10³ ± 1.59 × 10³	1.14 × 10³ ± 1.28 × 10³	0.123	206.85–1520.97
Biofilm	Baseline	3.77 × 10⁷ ± 1.36 × 10⁸	1.23 × 10⁴ ± 3.30 × 10⁴	0.442	0.915–1.967
	Day 4	2.12 × 10⁴ ± 5.68 × 10⁴	1.12 × 10⁴ ± 3.41 × 10⁴	0.806	0.465–0.586

Data are presented as mean ± standard deviation. Values are shown on the original scale; statistical analyses were performed using log-transformed data. p-values and 95% confidence intervals were calculated using two-way ANOVA with Bonferroni correction for multiple comparisons. Statistical significance was set at *p* < 0.05

**Table 11:** Intragroup comparison of *T. forsythia 16 S rRNA* gene copy numbers between baseline and day 4

Sample	Group	Baseline (copies/µL, Mean ± SD)	Day 4 (copies/µL, Mean ± SD)	*p*-value	95% CI
Saliva	Traditional MB	1.57 × 10³ ± 1.09 × 10³	1.88 × 10³ ± 1.59 × 10³	0.538	764.78–1392.49
	New MB formulation	1.31 × 10³ ± 1.26 × 10³	1.14 × 10³ ± 1.28 × 10³	0.518	205.13–385.58
Biofilm	Traditional MB	3.77 × 10⁷ ± 1.36 × 10⁸	2.12 × 10⁴ ± 5.68 × 10⁴	0.621	1.254–2.014
	New MB formulation	1.23 × 10⁴ ± 3.30 × 10⁴	1.12 × 10⁴ ± 3.41 × 10⁴	0.881	1.127–1.297

Data are presented as mean ± standard deviation. Values are shown on the original scale; statistical analyses were performed using log-transformed data. p-values and 95% confidence intervals were calculated using two-way ANOVA with Bonferroni correction for multiple comparisons. Statistical significance was set at *p* < 0.05

Regarding total bacterial counts in saliva, no statistically significant differences were found between or within groups at baseline or four days after treatment (*p* = 0.908 and *p* = 0.392; *p* = 0.414 and *p* = 0.747, respectively). However, the analysis of the biofilm revealed a significant reduction in total bacterial counts in the group treated with the novel MB formulation (*p* < 0.001), which was not observed in the traditional MB group (*p* = 0.223). A statistically significant difference was also found between groups in the final values (*p* < 0.001). although no corresponding statistically significant differences were observed in the primary clinical outcomes. The following tables and figures summarize the data obtained. No adverse effects were reported during the study (Tables 12 and 13).

**Table 12:** Intragroup comparison of total bacterial *16 S rRNA* gene copy numbers between baseline and day 4

Sample	Group	Baseline (copies/µL, Mean ± SD)	Day 4 (copies/µL, Mean ± SD)	*p*-value	95% CI
Saliva	Traditional MB	9.53 × 10⁷ ± 1.37 × 10⁸	3.56 × 10⁸ ± 1.05 × 10⁹	0.414	0.205–0.403
	New MB formulation	9.68 × 10⁷ ± 1.23 × 10⁸	7.85 × 10⁷ ± 8.23 × 10⁷	0.747	0.301–0.411
Biofilm	Traditional MB	2.07 × 10⁷ ± 1.87 × 10⁷	5.06 × 10⁷ ± 9.63 × 10⁷	0.223	0.107–0.415
	New MB formulation	3.11 × 10⁷ ± 3.33 × 10⁷	2.41 × 10⁷ ± 1.88 × 10⁷	< 0.001	12,729,404.7–35,469,149.3

Data are presented as mean ± standard deviation. Values are shown on the original scale; statistical analyses were performed using log-transformed data. p-values and 95% confidence intervals were calculated using two-way ANOVA with Bonferroni correction for multiple comparisons. Statistical significance was set at *p* < 0.05

**Table 13:** Intergroup comparison of total bacterial *16 S rRNA* gene copy numbers at baseline and day 4

Sample	Time point	Traditional MB (copies/µL, Mean ± SD)	New MB formulation (copies/µL, Mean ± SD)	*p*-value	95% CI
Saliva	Baseline	9.53 × 10⁷ ± 1.37 × 10⁸	9.68 × 10⁷ ± 1.23 × 10⁸	0.908	0.373–0.410
	Day 4	3.56 × 10⁸ ± 1.05 × 10⁹	7.85 × 10⁷ ± 8.23 × 10⁷	0.392	0.297–0.718
Biofilm	Baseline	2.07 × 10⁷ ± 1.87 × 10⁷	3.11 × 10⁷ ± 3.33 × 10⁷	0.778	0.557–0.727
	Day 4	5.06 × 10⁷ ± 9.63 × 10⁷	2.41 × 10⁷ ± 1.88 × 10⁷	< 0.001	12,729,404.6–35,469,148.9

Data are presented as mean ± standard deviation. Values are shown on the original scale; statistical analyses were performed using log-transformed data. p-values and 95% confidence intervals were calculated using two-way ANOVA with Bonferroni correction for multiple comparisons. Statistical significance was set at *p* < 0.05

## Discussion

The present study demonstrated that both treatment protocols resulted in a statistically significant reduction in pain intensity (G1: *p* = 0.022; G2: *p* = 0.001), which was the primary outcome of the study. This fact suggests that aPDT combined with saline irrigation is effective at reducing pain in cases of pericoronitis. Elsadek et al. (2020) [26] also found a significant reduction in pain seven and 14 days after aPDT with methylene blue (0.0005%), saline irrigation, and gentle debridement. Although no significant difference was found between aPDT and conventional irrigation with debridement alone, the aPDT group exhibited greater reductions in *P. gingivalis*, *T. forsythia*, and TNF-α, highlighting potential microbiological and anti-inflammatory benefits. These findings raise the question of whether different laser parameters may enhance the clinical superiority of aPDT. Supporting this, Eroglu et al. (2018) [27] reported significantly greater pain reduction at seven days in patients treated with antibiotics plus aPDT using indocyanine green and an 810 nm laser compared to those treated with antibiotics alone, demonstrating the therapeutic potential of aPDT in managing pericoronitis.

A statistically significant improvement in mouth opening was found in both groups (G1: *p* < 0.001; G2: *p* = 0.002), which was one of the secondary outcomes of the study. To date, no studies in the literature have assessed mouth opening in patients with pericoronitis treated with aPDT. However, Sezer et al. (2012) [20] reported a significant improvement in mouth opening after seven days of treatment with 660 nm and 808 nm diode lasers as well as Nd: YAG laser, although no statistically significant differences were found between treatment modalities. Additionally, Mesquita-Ferrari et al. (2011) [28] demonstrated that low-level laser therapy reduced pro-inflammatory cytokine release in rats following muscle injury after seven days. These findings indicate the possibility that improvements in mouth opening could be attributed to the antimicrobial properties of aPDT, the photobiomodulation effects of laser, or a combination of both.

A statistically significant difference in mouth opening was observed between groups at baseline, with lower values in the group receiving the new formulation. Despite this initial difference, no significant differences between groups were observed over time, suggesting that baseline imbalance did not influence the outcome.

In the present study, no significant intra- or intergroup differences were found in facial edema after treatment. This finding is likely due to the inclusion of patients with early stage pericoronitis, which is typically not characterized by pronounced edema [29]. Cases in which edema was present and accompanied by purulent exudate near the third molar were immediately excluded from the study.

To assess the impact of oral health on quality of life, all participants completed the OHIP-14 questionnaire before and after treatment. No significant reduction in the total OHIP-14 score was found in either group, suggesting a possible lack of perceived improvement. To date, no studies have applied this instrument specifically to patients with pericoronitis treated with aPDT. However, Yurttutan et al. (2020) [30] and Passarelli et al. (2020) [31] found significant reductions in OHIP-14 scores in patients with pericoronitis undergoing periodontal scaling or tooth extraction, with assessments performed at one week, one month, three months, and six months. Unlike these studies, the present investigation used a shorter follow-up period, which may explain the absence of significant changes. Given that the OHIP-14 is not disease-specific and addresses the frequency of impairments over the previous six months, its sensitivity in capturing the transient nature of symptoms of pericoronitis may be limited. Therefore, we also performed a partial analysis focusing on the physical pain and physical disability domains, as performed previously by McNutt et al. (2008) [32] and Santos et al. (2020) [33]. However, no significant differences were found. The use of an alternative questionnaire assessing the intensity rather than the frequency of symptoms may have yielded different outcomes in this clinical context.

The microbiological analysis revealed the presence of *T. forsythia* in 100% of the saliva and biofilm samples, which is consistent with data from previous studies reporting the high prevalence of this species in cases of pericoronitis [2, 6, 7]. Sencimen et al. (2014) [2] also associated the presence of *T. forsythia* with key clinical signs and symptoms. Although both groups in our study exhibited clinical improvement in pain and mouth opening, no statistically significant reduction in *T. forsythia* was detected in saliva or biofilm after either treatment. *T. forsythia* is typically found in mature biofilms and deeper sulcus regions [34]. Due to the structural organization of biofilm, methylene blue may have had limited interaction with *T. forsythia*, which may explain its persistence. Kolbe et al. (2014) [35] also found no significant reduction in this species even after three and six months of aPDT combined with scaling in periodontal patients. This suggests that other bacterial species or inflammatory factors may contribute to the clinical manifestations of pericoronitis.

A reduction in total bacterial load in the biofilm was found in the experimental group, which involved the novel MB formulation (*p* < 0.001). This finding may be partially related to the presence of the surfactant, which could enhance the distribution and interaction of the photosensitizer with the biofilm. Additionally, the ability of the surfactant to form micelles can improve the solubility and stability of MB, potentially optimizing its photochemical activity. Surfactants may also interact with bacterial membranes, increasing permeability and facilitating the action of the photosensitizer Supporting this, Collina et al. (2018) [16] found that among several vehicles tested (water, saline, PBS, and urea), only 0.25% sodium dodecyl sulfate (SDS) improved the antimicrobial efficacy of methylene blue against *Candida albicans* in a planktonic culture. Likewise, Tortamano et al. (2020) [36] reported that methylene blue in SDS eliminated *P. gingivalis* ATCC 33,577 within one minute, whereas the phosphate-buffered formulation required five minutes. Alvarenga et al. (2019) [19] demonstrated that MB in SDS achieved significant microbial reduction in periodontal pockets after five minutes of irradiation in clinical settings, which was not observed with aqueous MB.

Only in the experimental group, using the new MB formulation, was a reduction in total bacterial load in the biofilm observed (*p* < 0.001). However, this finding should be interpreted with caution, as no superiority was observed in the primary clinical outcomes, including pain and mouth opening. These findings suggest a potential microbiological effect; however, in the absence of demonstrated clinical superiority, they should not be overinterpreted, and their clinical relevance remains uncertain. Possible explanations for this finding may be related to differences in the physicochemical properties of the formulation, such as improved distribution and interaction with the biofilm; however, these mechanisms should be interpreted as hypothetical and require further investigation.

Unlike the biofilm results, the salivary analysis revealed no reduction in total bacterial load in either treatment group. Previous studies have shown that a reduction in oral biofilm often leads to lower salivary bacterial counts, likely due to diminished microbial transfer from biofilm to saliva following broad-spectrum antimicrobial therapies [37, 38]. However, most of these studies involved periodontal patients treated at multiple sites with full-mouth scaling, which contrasts with the present study, in which aPDT was applied to a single site. This limited application may explain the lack of a reduction in salivary bacteria despite significant local biofilm reduction achieved with the novel MB formulation. Supporting this, Bargrizan et al. (2018) [39] reported a reduction in salivary *S. mutans* following aPDT when multiple oral surfaces were irradiated, including the tongue and buccal mucosa. These findings suggest that effective salivary microbial control may require multi-site treatment, irrespective of the antimicrobial strategy used. Future studies involving subgingival microbiome profiling could clarify which specific bacterial species are most affected by localized aPDT and enable a better understanding of the broader microbiological impact of this treatment modality.

Multiple cytokines in the gingival crevicular fluid (GCF) were analyzed using the Luminex™ system (TNF-α, IL-1β, IL-6, IL-8, IL-10, IL-12, and IL-17). Thus, only IL-1β and IL-8 were consistently detectable across all samples and included in the statistical analyses. IL-1β is a key pro-inflammatory mediator involved in vascular changes and immune cell recruitment [40]. Elevated levels of this cytokine have been associated with symptomatic third molars and periodontal disease [41, 42]. The significant reduction in IL-1β in the group treated with the novel MB formulation may be associated with the concurrent decrease in total bacterial load in the biofilm, as bacterial components, such as lipopolysaccharides, are known to trigger IL-1β release [43]. Previous studies also demonstrated reductions in IL-1β following aPDT combined with scaling [35], whereas studies without adjunctive therapy, such as that conducted by Giannopoulou et al. (2012) [44], reported no significant changes in cytokine levels, highlighting the importance of biofilm disruption for therapeutic success.

IL-8 is another cytokine often elevated in periodontal inflammation and plays a central role in neutrophil recruitment to the gingival sulcus [45]. The lack of a significant reduction in IL-8 in our study, despite elevated baseline levels, may be related to the timing of the reassessment. Previous investigations reported significant reductions in IL-8 only after longer follow-up times ranging from one week to three months [46], suggesting that our timeframe may have been insufficient to detect such changes.

The study demonstrated that at baseline, prior to treatment, no statistically significant differences were observed between groups for nearly all evaluated variables, indicating that the groups were generally comparable before intervention. Nevertheless, a statistically significant difference in mouth opening was observed between groups at baseline. This finding was likely influenced by the presence of an outlier in one group, which may have affected measures of central tendency and variability, particularly given the small sample size.

This baseline imbalance represents a potential confounding factor and should be considered when interpreting the results. However, no statistically significant differences between groups were observed over time for this outcome, suggesting a limited impact on the overall findings.

. Based on our results, we cannot attribute the observed benefits solely to aPDT, nor can we conclude that they are specifically related to one methylene blue formulation over the other. Therefore, any interpretation suggesting superiority of the new formulation should be made with caution.

Despite the robust study design, the follow-up period is a limitation. All outcomes were assessed at baseline and 4 days post-treatment. This relatively short follow-up period is a limitation of the study, as pericoronitis may be self-limiting [47, 48]. Longer follow-up periods may provide a more comprehensive evaluation. Although the timing of reassessment was based on the typical course of acute inflammatory responses, earlier evaluations could reflect the natural progression of the condition rather than the true effect of the intervention. Therefore, longer follow-up periods, such as at least 7 days, may provide a more comprehensive evaluation of treatment outcomes and help distinguish between the natural course of the condition and the effects of the intervention, particularly regarding microbiological and cytokine responses.

Second, the study design did not include additional control groups, such as irrigation alone or laser therapy without a photosensitizer, which limits the ability to isolate the specific effects of antimicrobial photodynamic therapy and the contribution of each component of the intervention.

Third, although randomization was performed, a baseline imbalance in mouth opening was observed, which may have influenced the results despite no differences over time. Therefore, these limitations should be considered when interpreting the findings, and future studies with longer follow-up periods and additional control groups are warranted to better establish the clinical effectiveness and mechanisms of action of these protocols.

From a clinical perspective, both treatment protocols resulted in comparable improvements in pain and mouth opening, which are the most relevant outcomes for patient-centered management of pericoronitis. Although the new methylene blue formulation demonstrated reductions in specific microbiological and immunological parameters, these findings were not associated with superior clinical outcomes. Therefore, the clinical advantage of the new formulation in routine practice remains uncertain.

Despite the limitations mentioned, the improvements in pain intensity and mouth opening add significant value to this study. The clinical improvements suggest that aPDT is a promising treatment for pericoronitis, irrespective of the MB formulation used. In clinical practice, many dentists tend to prescribe antibiotics to patients with pericoronitis, exposing them to potential side effects [49]. Our results demonstrate that aPDT, which is a simple, low-cost technique that does not induce bacterial resistance [8], merits further investigation to establish safe, effective clinical protocols in dentistry. Only the group treated with the MB formulation containing SDS exhibited a statistically significant reduction in IL-1β and total bacterial counts. It is not possible to conclude that this formulation is clinically superior to the conventional methylene blue formulation. Both groups experienced improvements in pain and mouth opening, with no statistically significant differences between treatments. Further studies with MB are warranted to determine whether compositional differences may, at some point, provide clinical advantages.

Both groups demonstrated significant improvements in pain and mouth opening following aPDT, with no statistically significant differences between them. Although reductions in IL-1β levels and total bacterial load were observed in the group treated with the new formulation, these findings were limited to specific outcomes and were not associated with superior clinical effects. Therefore, within the limitations of this study, no clinical superiority of the new formulation over the conventional methylene blue formulation was demonstrated. At present, these findings do not support a change in clinical practice.

## Supplementary Information

Below is the link to the electronic supplementary material.

Supplementary Material 1

Supplementary Material 2

## Data Availability

The datasets generated and/or analyzed during the current study are not publicly available due to ethical and privacy restrictions on patient data. However, fully anonymized raw data are provided as a supplementary Excel file submitted with this manuscript. Additional data may be available from the corresponding author upon reasonable request and with permission from the appropriate ethics committee.
